# Role of Brachytherapy Boost in Clinically Localized Intermediate and High-Risk Prostate Cancer: Lack of Benefit in Patients with Very High-Risk Factors T3b–4 and/or Gleason 9–10

**DOI:** 10.3390/cancers14122976

**Published:** 2022-06-16

**Authors:** Hideya Yamazaki, Gen Suzuki, Koji Masui, Norihiro Aibe, Daisuke Shimizu, Takuya Kimoto, Kei Yamada, Koji Okihara, Takashi Ueda, Tsukasa Narukawa, Takumi Shiraishi, Atsuko Fujihara, Ken Yoshida, Satoaki Nakamura, Takashi Kato, Yasutoshi Hashimoto, Haruumi Okabe

**Affiliations:** 1 Department of Radiology, Graduate School of Medical Science, Kyoto Prefectural University of Medicine, 465 Kajiicho Kawaramachi Hirokoji, Kamigyo-ku, Kyoto 602-8566, Japan; gensuzu@koto.kpu-m.ac.jp (G.S.); mc0515kj@koto.kpu-m.ac.jp (K.M.); a-ib-n24@koto.kpu-m.ac.jp (N.A.); dshimizu@koto.kpu-m.ac.jp (D.S.); t-kimoto@koto.kpu-m.ac.jp (T.K.); kyamada@koto.kpu-m.ac.jp (K.Y.); 2 Department of Urology, Graduate School of Medical Science, Kyoto Prefectural University of Medicine, 465 Kajiicho Kawaramachi Hirokoji, Kamigyo-ku, Kyoto 602-8566, Japan; kokihara@koto.kpu-m.ac.jp (K.O.); t-ueda@koto.kpu-m.ac.jp (T.U.); tnarukaw@koto.kpu-m.ac.jp (T.N.); takumi14@koto.kpu-m.ac.jp (T.S.); fujihara@koto.kpu-m.ac.jp (A.F.); 3 Department of Radiology, Kansai Medical University, Hirakata 573-1010, Japan; yoshidaisbt@gmail.com (K.Y.); satoaki@nakamura.pro (S.N.); 4 Department of Radiology, Ujitakeda Hospital, Uji-City, Kyoto 611-0021, Japan; hymed_housya@yahoo.co.jp (T.K.); yasu06340829@yahoo.co.jp (Y.H.); h-okabe@takedahp.or.jp (H.O.)

**Keywords:** radiotherapy, brachytherapy boost, very high-risk, T3b–4, Gleason 9–10, prostate cancer

## Abstract

**Simple Summary:**

In general, brachytherapy (BT) improves biochemical control in intermediate-to high-risk prostate cancer. We previously reported that importance of very high-risk factors (VHR: T3b–4 or Gleason score 9–10) and patients with double VHR (VHR-2) showed the worst prognosis among high-risk groups. We explored the role of BT-boost in patients with VHR and compared it to intermediate- and other high-risk groups. We confirmed that BT-boost improved prostate-specific antigen (PSA) control but resulted in equivalent overall survival rates for the intermediate- and high-risk groups, except for the patients with VHR. In the VHR-1 group (single VHR), BT-boost showed superior PSA control to conventional-dose RT (EQD2 ≤ 72 Gy) but not to the dose-escalated radiotherapy group (EQD2 ≥ 74 Gy). In the VHR-2 group, BT-boost did not improve the biochemical control rate of either Conv RT or DeRT. BT-boost showed no benefit over modern DeRT in the patients with VHR.

**Abstract:**

This study examined the role of brachytherapy boost (BT-boost) and external beam radiotherapy (EBRT) in intermediate- to high-risk prostate cancer, especially in patients with very high-risk factors (VHR: T3b–4 or Gleason score 9–10) as patients with double very high-risk factors (VHR-2: T3b–4 and Gleason score 9–10) previously showed worst prognosis in localized prostate cancer. We retrospectively reviewed multi-institutional data of 1961 patients that were administered radiotherapy (1091 BT-boost and 872 EBRT: 593 conventional-dose RT (Conv RT: equivalent to doses of 2 Gy per fraction = EQD2 ≤ 72 Gy) and 216 dose-escalating RT (DeRT = EQD2 ≥ 74 Gy). We found that BT-boost improved PSA control and provided an equivalent overall survival rate in the intermediate- and high-risk groups, except for patients within the VHR factor group. In the VHR-1 group (single VHR), BT-boost showed a superior biochemical control rate to the Conv RT group but not to the DeRT group. In the VHR-2 group, BT-boost did not improve outcomes of either Conv RT or DeRT groups. In conclusion, BT-boost showed no benefit to modern DeRT in the patients with VHR; therefore, they are not good candidates for BT-boost to improve outcome and may be amenable to clinical trials using multimodal intensified systemic treatments.

## 1. Introduction

Dose escalation in radiotherapy is an established strategy to improve the biochemical control rate in clinically localized through intermediate- and high-risk localized prostate cancer [1,2,3,4]. Brachytherapy boost (BT-boost) combined with external beam radiotherapy (EBRT) is a good option to elevate the prescribed dose of radiotherapy without increasing the toxicity due to the superior character of the BT; the rapid falloff of the dose gradient enables us to treat tumors at high doses while maintaining low doses to the surrounding organs at risk. Retrospective [4,5,6,7,8] and prospective analyses, including three randomized controlled trials [9,10,11,12], as well as meta-analysis, confirmed the benefit of biochemical control for BT-boost [13].

Recent exploration of risk stratification has introduced a new concept of very high-risk factors (VHR). The most widely used risk classification system in clinics is the National Comprehensive Cancer Network (NCCN) [14], in which the VHR includes a >4 biopsy cores with a Gleason score of 8–10, clinical stage T3b–T4 or primary Gleason score of 5, [14]. For risk stratification including VHR, summation of the number of VHRs (T3b–4 and Gleason score 9–10) was a useful system to identify the worst oncological population [15]. As data were scarce regarding the merits of dose escalation in patients with VHR, the present study examined the role of dose escalation with BT-boost in patients with intermediate- to high-risk cancer as well as VHR. In addition, the NCCN Clinical Practice Guidelines in Oncology stated that doses of ≤70 Gy in conventional fractions are not sufficient for the treatment of intermediate- or high-risk prostate cancer [14,15]. Therefore, we divided the control group into conventional dose (Conv RT, doses ≤ 72 Gy) and dose-escalated radiotherapy (DeRT, prescribed dose of ≥74 Gy, equivalent to doses of 2 Gy per fraction [EQD2]) groups.

Therefore, this study examined the role of BT-boost in patients with VHR for the prognostication of clinically localized high-risk prostate cancer.

## 2. Materials and Methods

### 2.1. Patients

We used freely available public data to analyze a large cohort of 1961 patients; among these, 1145 BT-boost (1091 patients administered high-dose BT (HDR-BT) boost from open data for public use [16] and 63 patients treated with low-dose BT (LDR-BT) boost at the Kyoto Prefectural Medical School) [17]. This study included a total of 809 patients administered EBRT (388 administered Conv RT as identified from open data) and 421 patients [16] administered DeRT using intensity-modulated radiotherapy [IMRT] from open data [16] at Uji Takeda Hospital [18] (Table 1). Patients eligible for this study were treated with EBRT with BT-boost or EBRT alone; had histology-proven adenocarcinoma with clinical TNM stage T1–T4N0M0 disease; and had accessible and available data on T classification, Gleason score sum, and initial PSA [iPSA] level. The patients categorized as intermediate or high risks were eligible according to the NCCN risk classification [14]. A simple VHR index was calculated and applied by summing the number of VHR factors in the high-risk group: VHR-0, no VHR; VHR-1, Gleason score 9–10 or T3b–T4; VHR-2, Gleason score = 9–10 and T3b–T4 [19].

We defined PSA failure according to the Phoenix definitions (nadir, +2 ng/mL).

Prostate cancer-specific mortality (PCSM) was defined when prostate cancer was the primary cause of death. Biochemical disease-free survival (bDFS), PCSM, overall survival (OS), and metastasis-free survival (MFS) rates were defined as the intervals from the start of RT to bDFS, distant metastasis, PCSM, and death, respectively.

Patients included in the public data provided informed consent during the process of building the database and all patients from Uji Takeda Hospital and Kyoto Prefectural Medical School provided written informed consent [19]. This study was approved by the Institutional Review Board of the Kyoto Prefectural University of Medicine (ERB-C-1403) and conducted by the principles of the Declaration of Helsinki.

### 2.2. Treatment Planning

#### 2.2.1. BT-Boost

The BT-boost groups included HDR-BT and LDR-BT. A multi-institution data of HDR-BT was provided from an open data source [16], and details of treatment have been described elsewhere [20,21]. In brief, the median dose of HDR-BT was 31.5 Gy (range, 10.5–31.5 Gy) in median fraction size 6.3 Gy (range, 5–11 Gy) combined with EBRT in various dose and fractions (median 3 Gy; range, 1.9–3.1 Gy) (Supplemental Table S1). The detailed treatment schedule for LDR-BT (Iodine-125 implantation) was described previously [17]. We included patients with T3a disease or Gleason score sum ≤ 8 or a summed Gleason score of 7 (4 + 3), but not for those with a summed Gleason score of 7 (3 + 4) [17] using prescription dose 110 Gy (LDR-BT) with EBRT by three-dimensional conformal radiotherapy (3D-CRT) 40 Gy/20 fractions (Supplemental Table S1). Whole pelvic RT were used in several institutions as a part of EBRT (Supplemental Table S1).

#### 2.2.2. External Beam Radiotherapy (EBRT)

The EBRT group consisted of conventional two-dimensional treatment, 3D-CRT, and IMRT. Supplemental Table S1 depicted the details of patient backgrounds. A freely accessible dataset (*n* = 417) was used to draw some of EBRT data [16]; 141 image-guided IMRTs using helical TomoTherapy were performed at the Department of Radiology, Uji Takeda Hospital, and detailed technique has been described elsewhere [18]. In brief, the prescribed dose was 74 Gy/37 fractions (2 Gy/fraction, *n* = 79) or 74.8 Gy/34 fractions (2.2 Gy/fraction, *n* = 62) for the intermediate-risk and high-risk and groups [18]. We divided a control group into conventional dose group (Conv RT) using does up to 72 Gy and dose-escalated radiotherapy (DeRT) using dose 74 Gy or more in equivalent to doses of 2 Gy per fraction (EQD2). Detail of treatment schedules was depicted in Supplemental Table S1.

### 2.3. Statistical Analysis

EZR stat package [22] and StatView 5.0 (SAS Institute, Inc., Cary, NC, USA) were used to perform the statistical analyses. Percentages were analyzed using Fisher’s exact tests for two groups and chi-square tests for three or more groups. To compare means or medians, Student *t*-tests were used for normally distributed data, and Mann–Whitney U- and Kruskal–Wallis tests for skewed data (i.e., PSA values) [22]. To analyze the biochemical disease-free survival rate (bDFS), distant metastasis-free survival (DMSF), overall survival (OS), and prostate cancer-specific survival rate (PCS), the Kaplan–Meier method was used. Log-rank tests and Bonferroni correction comparisons were performed in analysis of statistically significance. Cox’s proportional hazard model for bDFS was used for univariate and multivariate analyses. Statistical significance was set at *p* < 0.05. The propensity score was the probability of being assigned to each group and was calculated using a logistic regression model constructed with the baseline covariates shown in Table 2 (age, T classification, GS, pretreatment PSA, and hormonal therapy history). We used propensity score matching to reduce the selection bias for BT-boost or EBBT (a 1:1 matched cohort was made for comparison of BT-boost and EBRT in the total population and BT-boost versus DeRT).

## 3. Results

### 3.1. Patient and Disease Characteristics

All 1961 patients with intermediate-to-high-risk prostate cancer were treated with either BT-boost (*n* = 1152) or EBRT (*n* = 809). The median patient age was 71 years (range, 49–89 years). The median initial PSA value was 14.0 ng/mL (range, 2.682–1454 ng/mL). The clinical characteristics of the patients are summarized in Table 1. The median follow-up duration was 69.0 (range: 2–177) months.

### 3.2. Biochemical Control Rates (Biochemical Disease-Free Survival Rate; bDFS)

**Figure 1:**
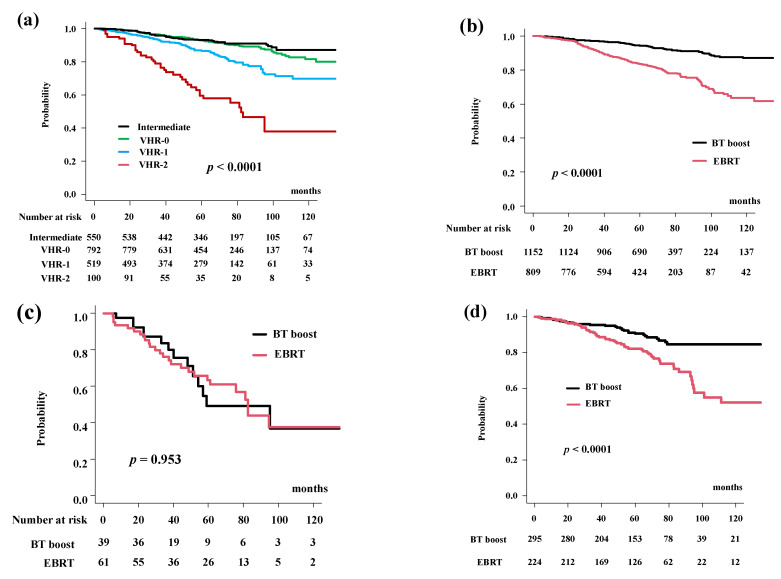

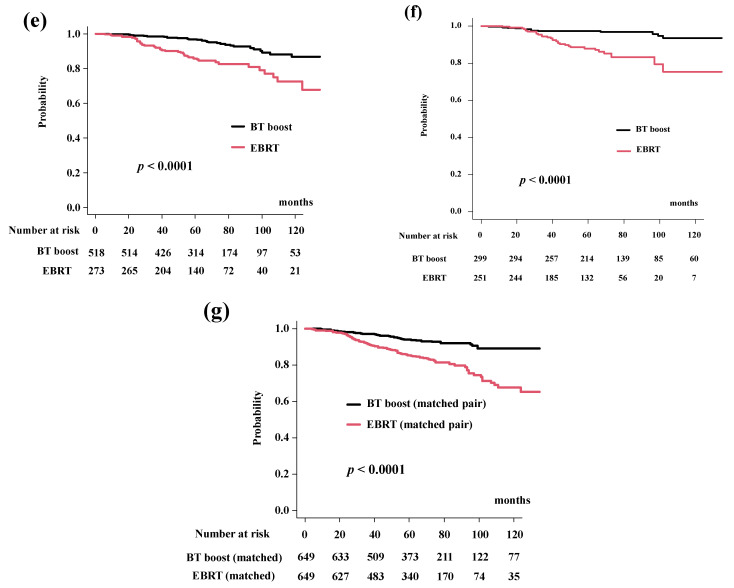
Biochemical control rates (biochemical disease-free survival rate; bDFS). (**a**) bDFS according to risk stratification. bDFS rates were 59.6% (95% CI: 47.5–69.7%), 86.8% (95% CI: 83.1–89.7%), 93.0% (95% CI: 90.8%–94.8%), 93.3% (95% CI: 90.7%–95.2%) at 5 years in the VHR-2, VHR-1, VHR-0, and intermediate groups, respectively (*p* < 0.0001). (**b**) Comparison of bDFS between EBRT and BT-boost. (**c**) Comparison of bDFS between EBRT and BT-boost in VHR-2 group. (**d**) Comparison of bDFS between EBRT and BT-boost in VHR-1 group. (**e**) Comparison of bDFS between EBRT and BT-boost in VHR-0 group, (**f**) Comparison of bDFS between EBRT and BT-boost in intermediate-risk group. (**g**) Comparison of bDFS between EBRT and BT-boost using matched pair analysis.

The actuarial 5-year bDFS rates were 89.9% (95% confidence interval [CI]: 88.3–91.2%) at 5 years and 78.2% (95% CI: 74.9–81.2%) at 10 years. The bDFS differed significantly among the four risk groups (*p* < 0.0001; Figure 1a).

BT-boost improved the bDFS to 94.2% (95% CI: 92.5–95.5%) at 5 years and 86.9% (95% CI: 83.4–89.8%) at 10 years compared to those in the EBRT group (83.7%, 95% CI: 80.7–86.3% at 5 years and 63.7%, 95% CI: 56.8–69.7% at 10 years) (Figure 1b, *p* < 0.0001).

The BT-boost group showed superior bDFS compared to that for EBRT in all groups, except for the VHR-2. The BT-boost group had bDFS rates of 49.1% (95% CI: 27.0–67.9%), 90.5% (95% CI: 85.8–93.8%), 96.9% (95% CI: 94.7–98.1%), 97.3% (95% CI: 94.6–98.6%) at 5 years in the VHR-2, VHR-1, VHR-0, and intermediate groups, respectively. The EBRT group had bDFS rates of 63.4% (95% CI: 48.8–74.8%, *p* = 0.953, Figure 1c), 82.2% (95% CI: 76.1–86.9%, *p* < 0.0001, Figure 1d), 85.8% (95% CI: 80.5–89.7%, *p* < 0.0001, Figure 1e), 87.9% (95% CI: 82.5–91.7%, *p* < 0.0001, Figure 1f) at 5-years in the VHR-2, VHR-1, VHR-0, and intermediate groups, respectively.

We applied propensity score matching to generate well-matched pairs (649 and 649 patients; background comparisons are shown in Supplemental Table S1b). The actuarial 5-year biochemical control rates were 93.9% (95% CI: 91.5–95.7%) and 85.3% (95% CI: 82.0–88.0%, *p* < 0.0001, Figure 1g) in the BT-boost and EBRT groups, respectively.

### 3.3. Subgroup Analysis (DeRT, Conv RT vs. BT-Boost)

**Figure 2:**
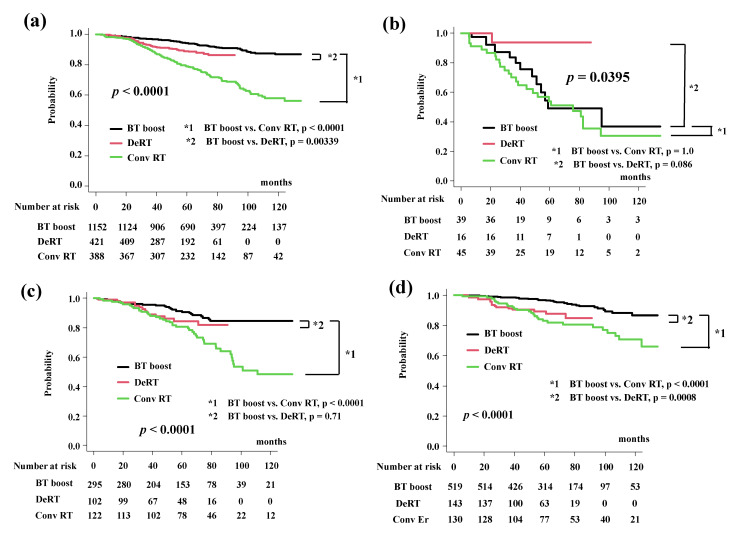

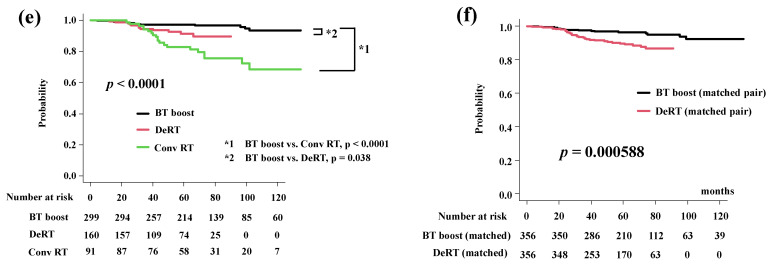
Biochemical control rates among three groups (BT-boost vs. Conv RT vs. DeRT). (**a**) Comparison of bDFS among three groups. (**b**) Comparison of bDFS among three groups in VHR-2 group. (**c**) Comparison of bDFS among three groups in VHR-1 group. (**d**) Comparison of bDFS among three groups in VHR-0 group. (**e**) Comparison of bDFS among three groups in intermediate-risk group. (**f**) Comparison of bDFS between dose escalated radiotherapy (DeRT) and BT-boost using matched pair analysis. bDFS = biochemical disease-free survival rate.

After dividing EBRT into the Conv RT and DeRT groups (Table 2), the bDFS for each group were 94.2% (95% CI: 92.5%–95.5%), 89.1% (95% CI: 85.2–92.0%), and 79.0% (95% CI: 74.3–82.9%) at 5 years and 86.9% (95% CI: 83.4–89.8%), not available (86.4% at 74 months; 95% CI: 81.5–90.0%), 58.0% (95% CI: 50.9–64.5) at 10 years in the BT-boost group, DeRT and Conv RT group (Figure 2a). BT-boost showed the best outcome among the three groups, with a statistically significant difference not only between the Conv and BT-boost groups but also between the DeRT and BT-boost groups (Figure 2a).

In detailed analysis, BT-boost showed the better outcome not only than Conv RT but also DeRT, except in patients with VHR. The BT-boost group had bDFS rates of 49.1% (95% CI: 27.0–67.9%), 90.5% (95% CI: 85.8–93.8%,), 96.9% (95% CI: 94.7–98.1%), and 97.3% (95% CI: 94.6–98.6%) at 5 years for the VHR-2, VHR-1, VHR-0 and intermediate groups, respectively, while the Conv RT and DeRT group had rates of 54.2% (95% CI: 37.7–68.0%, *p* = 1.0 in comparison to BT-boost, Figure 2b), 80.8% (95% CI: 72.2–86.9%, *p* < 0.0001, Figure 2c), 83.2% (95% CI: 74.8–89.0%, *p* < 0.0001, Figure 2d), 82.9% (95% CI: 72.9–89.5%, *p* < 0.0001, Figure 2e) and 93.7% (95% CI: 63.2–99.1%, *p* = 0.086, Figure 2b), 84.5% (95% CI: 74.6–90.8%, *p* = 0.71, Figure 2c), 89.2% (95% CI: 82.3–93.5%, *p* = 0.0008, Figure 2d), 91.4% (95% CI: 84.8–95.3%, *p* = 0.038, Figure 2e), at 5 years. We generated well-matched pairs for the comparison between BT-boost and DeRT (356 patients each; the background comparisons are shown in Supplemental Table S2) using propensity score matching. The actuarial 5-year biochemical control rates in the BT-boost and DeRT groups were 96.3% (95% CI: 93.5–97.9%) and 89.6% (95% CI: 85.5–92.6%, *p* = 0.000588, Figure 2f), respectively.

As shown in Table 3, the predictors of biochemical control on multivariate analysis included age, treatment modality (BT-boost vs. EBRT or DeRT or Conv RT), iPSA, T classification, and Gleason score sum.

### 3.4. Distant Metastasis-Free Survival (DMFS) Rates

**Figure 3:**
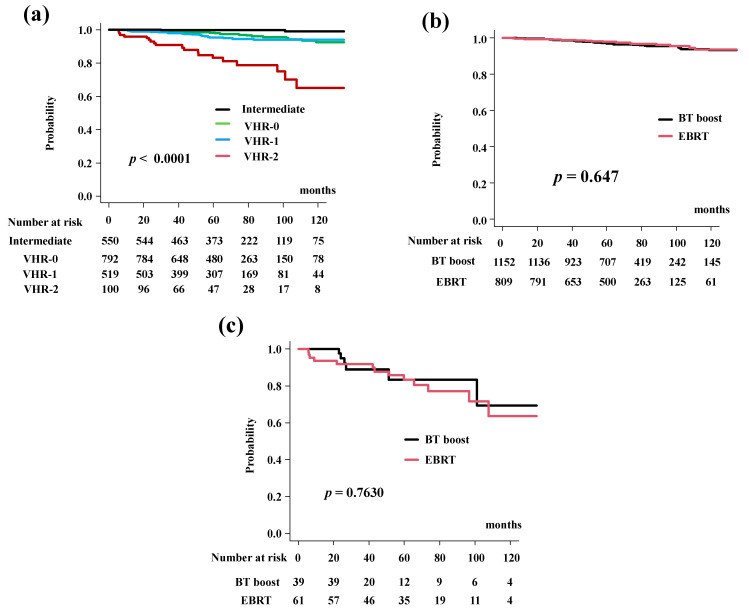
Distant metastasis-free survival rate (DMFS). (**a**) DMFS according to risk stratification. DMSF were 83.2% (95% CI: 72.9–89.8%), 95.4% (95% CI: 92.9–97.1%), 98.2% (95% CI: 96.8%–99.0%), 99.8% (95% CI: 94.8%–100%) at 5 years in the VHR-2, VHR-1, VHR-0, and intermediate groups, respectively (*p* < 0.0001). (**b**) Comparison of DMFS between EBRT and BT-boost. (**c**) Comparison of DMFS between EBRT and BT-boost in VHR-2 group.

The DMFS rates were 97.2% (95% CI: 96.3–97.9%) at 5 years and 93.3% (95% CI: 90.9–95.0%) at 10 years. The BT-boost group had DMSF rates of 96.9% (95% CI: 95.5–97.8%) at 5 years and 93.1% (95% CI: 90.1–95.2%) at 10 years. The DMFS rates differed significantly among the four risk groups (*p* < 0.0001; Figure 3a). The EBRT group had PCSM rates of 97.7% (95% CI: 96.2–98.6%) at 5 years and 93.4% (95% CI: 88.9%–96.1%) at 10 years (*p* = 0.647, Figure 3b).

The BT-boost group showed DMSF equivalent to EBRT in all groups. The BT-boost group had DMSF rates of 96.9% (95% CI: 62.7–93.2%), 93.7% (95% CI: 89.5–96.3%), 97.7% (95% CI: 95.6–99.7%), 99.7% (95% CI: 97.6–100%) at 5 years in the VHR-2, VHR-1, VHR-0 and intermediate groups, respectively, while the rates in the EBRT were 83.3% (95% CI: 70.1%–91.0%, *p* = 0.7630, Figure 3c), 97.4% (95% CI: 93.8–98.9%, *p* = 0.0696, Supplemental Figure S1a), 99.6% (95% CI: 97.4–99.9%, *p* = 0.3840, Supplemental Figure S1b), and 100% (*p* = 0.304, Supplemental Figure S1c) at 5 years.

### 3.5. Prostate Cancer-Specific Mortality (PCS)

**Figure 4:**
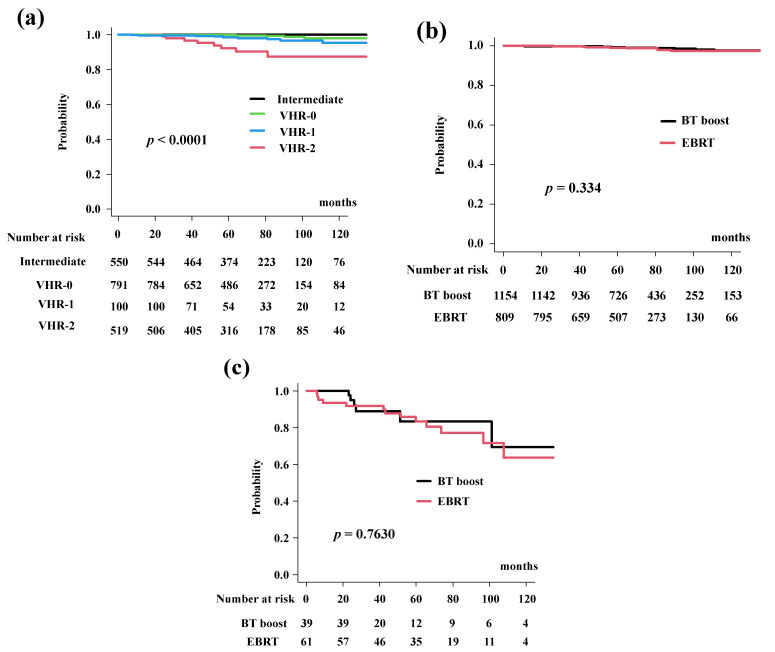
Prostate cancer specific survival rate (PCS). (**a**) PCS according to risk stratification. PCS were 93.8% (95% CI: 85.6–97.4), 98.5% (95% CI: 96.6–99.3%), 99.9% (95% CI: 98.5%–99.9%), and 100% at 5 years in the VHR-2, VHR-1, VHR-0, and intermediate groups, respectively (*p* < 0.0001). (**b**) Comparison of PCS between EBRT and BT-boost. (**c**) Comparison of PCS between EBRT and BT-boost in VHR-2 group. The BT-boost group showed equivalent PCS to EBRT in all groups.

The cumulative incidence for PCS was 99.2% (95% CI: 98.6–99.5%) at 5 years and 97.5% (95% CI: 95.9–98.5%) at 10 years in the total population. The BT-boost group in the present study had PCS rates of 99.2% (95% CI: 98.4–99.6%) at 5 years and 97.6% (95% CI: 95.4–98.8%) at 10 years. The PCS rates in the EBRT group were 99.1% (95% CI: 98.0–99.6%) at 5 years and 97.4% (95% CI: 94.9–98.6%) at 10 years (Figure 4a, *p* = 0.334). The PCS rates differed significantly among the four risk groups (*p* < 0.0001; Figure 4b).

In detail, the BT-boost group had PCS rates of 92.3% (95% CI: 70.6–98.2%), 97.6% (95% CI: 64.2–99.0%), 100%, and 100% at 5 years for VHR-2, VHR-1, VHR-0 and intermediate groups, while the EBRT group had a PCS of 92.2% (95% CI: 80.4–97.0%, Figure 4c), 99.5% (95% CI: 96.3–99.9%, *p* = 0.499, Supplemental Figure S2a), 99.6% (95% CI: 97.4–99.9%, *p* = 0.877, Supplemental Figure S2b), and 100% at 5-years (Supplemental Figure S2c).

### 3.6. Overall Survival (OS)

**Figure 5:**
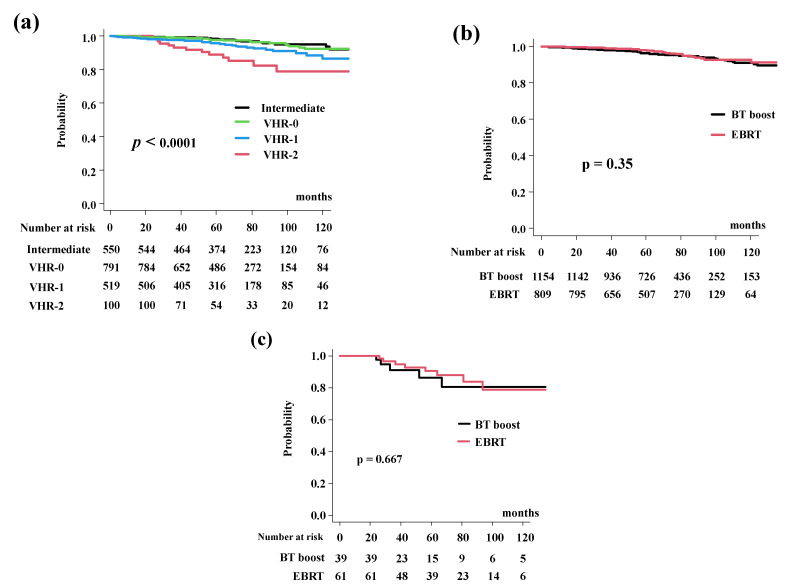
Overall survival rate (OS). (**a**) OS according to risk stratification. OS were 89.0% (95% CI: 79.7–94.2%), 95.9% (95% CI: 93.6–97.4%), 97.6% (95% CI: 96.1%–98.6%), 98.5% (95% CI: 96.9%–99.3%) at 5 years in the VHR-2, VHR-1, VHR-0, and intermediate groups, respectively (*p* < 0.0001). (**b**) Comparison of bDFS between EBRT and BT-boost. (**c**) Comparison of bDFS between EBRT and BT-boost in VHR-2 group. The BT-boost group showed an equivalent OS to those for EBRT in all groups.

The BT-boost group showed equivalent OS rates of 96.3% (CI: 94.9–97.3%) at 5 years and 91.0% (95% CI: 87.8–93.4%) at 10 years. In the EBRT group, the OS was 98.0% (95% CI: 96.6%–98.9%) at 5 years and 92.8% (95% CI: 89.2–95.2%) at 10 years (Figure 5a, *p* = 0.35). The OS differed significantly among the four risk groups (*p* < 0.0001; Figure 5b).

In detail, the BT-boost group had an OS of 86.3% (95% CI: 66.4–94.8%), 93.6% (95% CI: 89.6–96.1%), 97.6% (95% CI: 95.6–98.7%). and 97.7% (95% CI: 95.0–99.0%), at 5 years for the VHR-2, VHR-1, VHR-0, and intermediate groups. The EBRT group had OS rates of 90.5% (95% CI: 78.5–95.9%, *p* = 0.667, Figure 5c), 98.9% (95% CI: 95.5–99.7%, *p* = 0.177, Supplemental Figure S3a), 97.7% (95% CI: 994.6–99.1%, *p* = 0.322, Supplemental Figure S3b), and 99.6% (95% CI: 97.2–99.9%. *p* = 0.587, Supplemental Figure S3c), at 5-years.

## 4. Discussion

The present study explored the role of BT-boost in intermediate- to high-risk prostate cancer. The results demonstrated that BT-boost showed superior bDFS compared to that in the EBRT group except for the VHR-2 group. To our knowledge, this is the first report to show the merits and limitations of BT-boost in patients with intermediate-to high-risk prostate cancer, with a focus on the VHR-2 group. In their meta-analysis of three randomized control trials (RCTs), Kee et al. reported a significant benefit in 5-year bDFS in favor of BT-boost versus EBRT but not in OS and grade ≥ 3 late toxicities [12]. However, two of the RCTs [9,10,11] had a major bias in their methodologies, as the EBRT arm was not the standard care of treatment (too few doses were delivered in the EBRT arm) and their findings could not be translated into modern clinical situations. However, the recent Androgen Suppression Combined with Elective Nodal and Dose Escalated Radiation Therapy (ASCENDE-RT) trial showed that even with escalated EBRT, BT-boost provided better benefits in terms of biochemical control [9]. Our data were also consistent with their data demonstrating superior bDFS for BT-boost compared to both Conv RT and DeRT in patients without VHR. The use of androgen deprivation therapy (ADT) and its optimal duration was another confounding factor for bDFS analysis. The bDFS was significantly better regardless of the BT technique used (low or high dose rates) in these three RCTs, independent of the ADT duration. Higher BED delivered by BT (12–38%) with the better dose distribution due to the steep dose gradient, which delivered a non-homogeneous dose escalation [12,13], was an important factor to improve the outcomes. Furthermore, several studies have reported the superior efficacy of BT-boost not only in terms of bDFS but also in PCS and OS [7,8]. Therefore, BT-boost for intermediate- and high-risk prostate cancer was an attractive technique in numerous retrospective and prospective studies.

The concept of VHR was recently introduced. High-risk prostate cancer has been subdivided according to VHR in several ways. The NCCN used clinical stage T3b–T4 lesions, primary Gleason score = 5, or > 4 biopsy cores with Gleason scores of 8–10 [14]. After the initial estimation [23], a confirmation study was conducted, despite different definitions of VHR [14,24,25]. We also confirmed the importance of VHR factors, in which VHR-2 showed a higher hazard risk for DMSF, PCS, and OS than VHR-0 (hazard ratio = 8.81, 11.99, and 4.644, respectively) and VHR-1 (hazard ratio = 5.268, 2.359, and 2.896, respectively), and was a potentially better stratification system than the previous ones [15]. Our results add additional evidence of VHR-2 in a population at very high risk for recurrence outside the prostate (i.e., distant metastasis), even with the highest intensification of local radiotherapy with BT-boost. Our data could provoke a controversy regarding the indication for BT-boost in the patients with VHR, who may not be good candidates for BT-boost. These VHR criteria may be beneficial for better treatment choice for individual patients according to prognosis of the high-risk disease predisposing a risk of aggressive oncological outcomes, which may require intensive follow-up for metastasis using modern technologies; prostate-specific membrane antigen positron emission tomography scan [26] and earlier and/or adjuvant systemic therapy; or longer periods of ADT use in addition to abiraterone, docetaxel, and enzalutamide [27,28,29,30], which could be in a multimodal treatment clinical trial setting.

The present study has several limitations. First, the role of the biopsy core in the VHR system could not be analyzed because the public database did not contain these data. In addition, recent image-guided biopsy techniques made it impossible to assess older data as it was not compatible with recent systems. Second, the retrospective nature, limited follow-up time, and small sample size (especially in the VHR-2 group) in this study may limit the application of its findings. Thus, studies with longer follow-up and larger samples are needed to obtain concrete conclusions; although it could be difficult to perform, an RCT is anticipated.

## 5. Conclusions

BT-boost improved bDFS in intermediate- and higher risk groups, except for the patients with very high-risk factors (VHR-2: T3b-4 and Gleason 9–10). In the VHR-1 group (single VHR), BT-boost showed a superior biochemical control rate to the Conv RT group but not the DeRT group. In the VHR-2 group (double VHR), BT-boost did not improve outcomes of either the Conv RT or DeRT group. BT-boost showed no benefit to modern DeRT in the patients with VHR; therefore, they are not good candidates for BT-boost to improve outcome and may be amenable to clinical trials using multimodal intensified systemic treatments.

## Figures and Tables

**Table 1:** Patients’ characteristics.

Factor	Group	Subgroup	BT Boost	EBRT	p-Value
			n = 1152	n = 809	
Age			70 [49, 86]	72 [52, 89]	***<0.001***
iPSA (ng/mL)			13.60 [2.68, 500.00]	14.49 [2.91, 1454.00]	0.087
Gleason score	≤ 6		93 (8.1)	76 (9.4)	0.5443
	7		566 (49.1)	403 (49.8)	
	8 ≤		493 (42.8)	330 (40.8)	
T	1		242 (21.0)	176 (21.8)	***0.031***
	2		418 (36.3)	293 (36.3)	
	3		483 (41.9)	319 (39.5)	
	4		9 (0.8)	19 (2.4)	
NCCN risk classification	Intermediate-risk		299 (25.9)	251 (31.0)	***<0.001***
	High-risk	VHR-0: No T3b-4 nor G9–10	519 (45.1)	273 (33.7)	
		VHR-1: T3b-4 or G9–10	295 (25.6)	224 (27.7)	
		VHR-2: T3b-4 and G9–10	39 (3.4)	61 (7.5)	
Modality	BT-boost	LDR-BT	61 (5.3)	0 (0.0)	***<0.001***
		HDR-BT	1091 (94.5)	0 (0.0)	
	EBRT	DeRT (High BED)	0 (0.0)	421 (52.0)	
		Conv RT (Low BED)	0 (0.0)	388 (48.0)	
ADT	Yes		1076 (93.2)	683 (84.4)	***<0.001***
	No		78 (6.8)	126 (15.6)	
	Duration	(month)	42.00 [0.00, 128.00]	8.00 [0.00, 140.00]	***<0.001***
Follow-up		(month)	70.00 [2.00, 177.00]	68.00 [6.37, 145.63]	0.083

EBRT = external beam radiotherapy. De RT; Dose-escalated radiotherapy = EQD2 ≥ 74 Gy. Conv RT; Conventional radiotherapy = EQD2 ≤ 72 Gy.

**Table 2:** Detailed patient characteristics among subgroups.

			BT Boost		EBRT	
Factor	Group	Subgroup	HDR + EBRT	LDR + EBRT	Conv RT	DeRT
			n = 1091	n = 61	n = 593	n = 216
Age			70.00 [49.00, 86.00]	68.00 [52.00, 79.00]	72.00 [52.00, 89.00]	72.00 [54.00, 86.00]
T1234 (%)	1		230 (21.1)	12 (19.7)	121 (20.5)	55 (25.5)
	2		379 (34.7)	39 (63.9)	194 (32.8)	99 (45.8)
	3		473 (43.4)	10 (16.4)	258 (43.7)	61 (28.2)
	4		9 (0.8)	0 (0.0)	18 (3.0)	1 (0.5)
iPSA (ng/ml)			14.39 [2.68, 500.00]	7.70 [3.20, 46.00]	16.83 [2.91, 1454.00]	11.18 [4.00, 265.00]
Gleason score	−6		84 (7.7)	9 (14.8)	41 (6.9)	35 (16.2)
	7		534 (48.9)	32 (52.5)	325 (54.8)	78 (36.1)
	8−		473 (43.4)	20 (32.8)	227 (38.3)	103 (47.7)
ADT (%)	Yes		1041 (95.4)	26 (42.6)	522 (88.0)	161 (74.5)
	No		50 (4.6)	35 (57.4)	71 (12.0)	55 (25.5)
ADT duration	(months)		43.00 [0.00, 128.00]	5.00 [0.00, 14.00]	9.00 [0.00, 140.00]	6.00 [0.00, 80.00]
Risk classification (%)	Intermediate		269 (24.7)	30 (49.2)	176 (29.7)	75 (34.7)
	High		822 (75.3)	31 (50.8)	417 (70.3)	141 (65.3)
		VHR-0	487 (59.3)	31 (50.8)	199 (47.7)	74 (52.5)
		VHR-1	295 (35.9)	0 (0.0)	165 (39.6)	59 (41.8)
		VHR-2	39 (4.8)	0 (0.0)	53 (12.7)	8 (5.7)
Follow-up	(months)		68.00 [2.00, 177.00]	78.00 [17.00, 148.00]	60.37 [6.37, 145.63]	74.00 [23.17, 92.67]

**Table 3:** Multivariate analysis for PSA control.

Factor	Strata	Hazard Ratio	*p*-Value
Age	Sequential value	0.97 (0.95–1.00)	***0.028***
BT-boost vs. EBRT	BT-boost vs. EBRT	2.83 (2.15–3.72)	***<0.0001***
	BT-boost vs. DeRT	2.05 (1.40–3.01)	***0.00023***
	BT-boost vs. Conv RT	3.32 (2.47–4.45)	***<0.0001***
iPSA	0–9.9 vs. 10–20 vs. 20.1-(ng/mL)	1.33 (1.11–1.59)	***0.0018***
T classification	T1 vs. 2 vs. 3 vs. 4	1.38 (1.14–1.66)	***0.0009***
Gleason score sum	GS-6 vs. 7 vs. 8-	1.57 (1.25–1.97)	***0.00011***
ADT usage	Yes vs. No	0.66 (0.39–1.11)	*0.12*
Age	Sequential value	0.97 (0.95–1.00)	***0.028***

## Data Availability

The data of HDR-BT and part of EBRT for this manuscript can be obtained from the public database [19] and another part of EBRT and LDR-BT can be obtained from the author upon reasonable request.

## Supplementary Materials

The following are available online at https://www.mdpi.com/article/10.3390/cancers14122976/s1, Figure S1: Distant metastasis-free survival rate (DMFS), (**a**) Comparison of DMFS between EBRT and BT-boost in VHR-1 group. (**b**) Comparison of DMFS between EBRT and BT-boost in VHR-0 group. (**c**) Comparison of DMFS between EBRT and BT-boost in intermediate-risk group. Figure S2: Prostate cancer specific survival rate (PCS), (**a**) Comparison of PCS between EBRT and BT-boost in VHR-1 group. (**b**) Comparison of PCS between EBRT and BT-boost in VHR-0 group. (**c**) Comparison of PCS between EBRT and BT-boost in intermediate-risk group. Figure S3. Overall survival rate (OS), (**a**) Comparison of bDFS between EBRT and BT-boost in VHR-1 group. (**b**) Comparison of bDFS between EBRT and BT-boost in VHR-0 group. (**c**) Comparison of bDFS between EBRT and BT-boost in intermediate-risk group. Table S1: Detailed treatment schedule, Table S2: Comparison of background of Patient characteristics after propensity score matching, Table S3: Background Comparison between BT-boost and DeRT after propensity score matching.
