# Supplementary material for: Role of Brachytherapy Boost in Clinically Localized Intermediate and High-Risk Prostate Cancer: Lack of Benefit in Patients with Very High-Risk Factors T3b–4 and/or Gleason 9–10
Source: Cancers (Basel). 2022 Jun 16;14(12):2976. doi: 10.3390/cancers14122976 (PMC9221358; doi:10.3390/cancers14122976)
Supplement: Supplementary file 1 [file cancers-14-02976-s001.zip › cancers-1752125-supplementary/Supplemental Figures .pptx]

## Slide 1
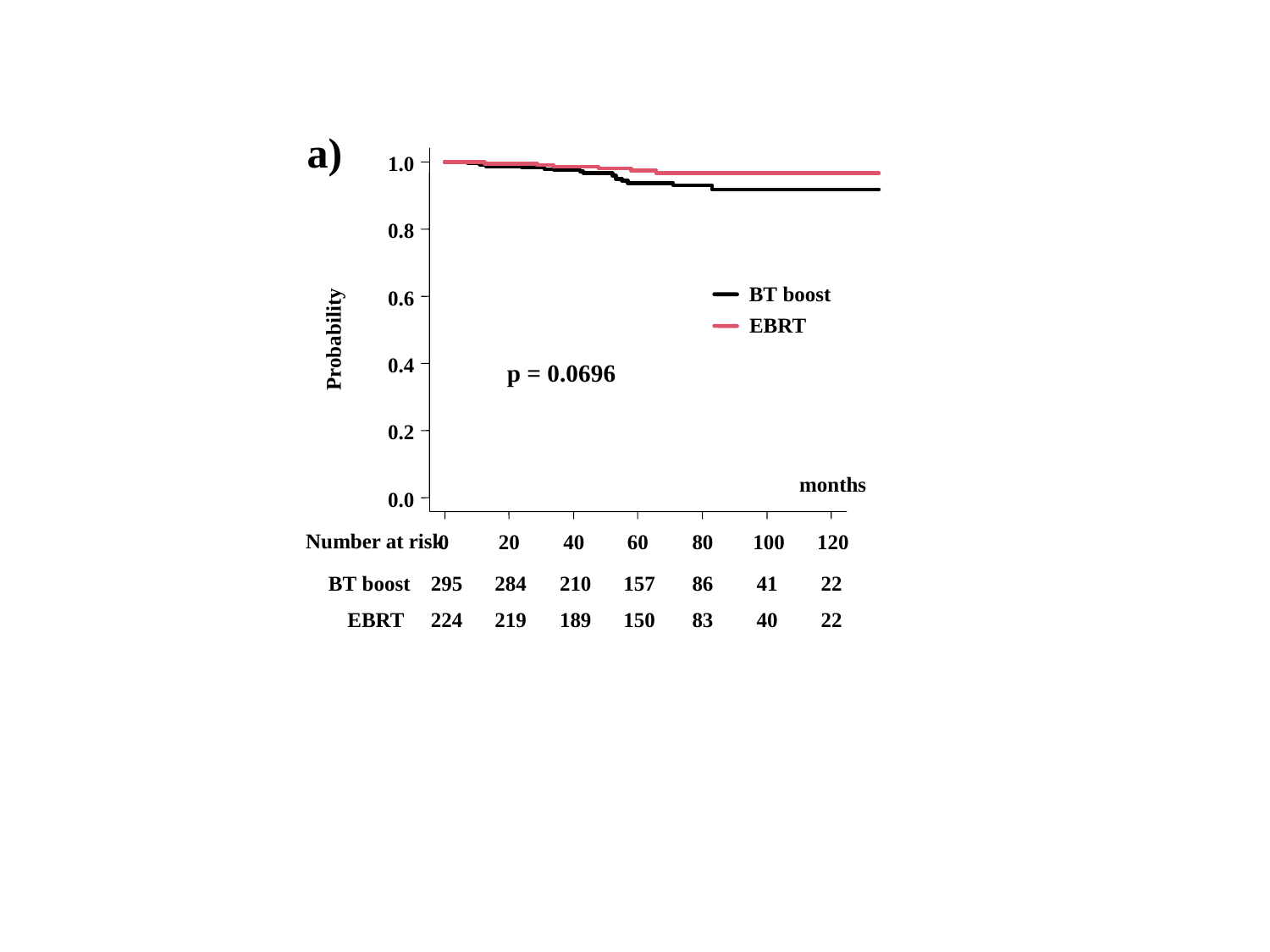

a)
1.0
0.8
BT boost
0.6
EBRT
Probability
p = 0.0696
0.4
0.2
months
0.0
Number at risk
0
20
40
60
80
100
120
BT boost
295
284
210
157
86
41
22
EBRT
224
219
189
150
83
40
22

## Slide 2
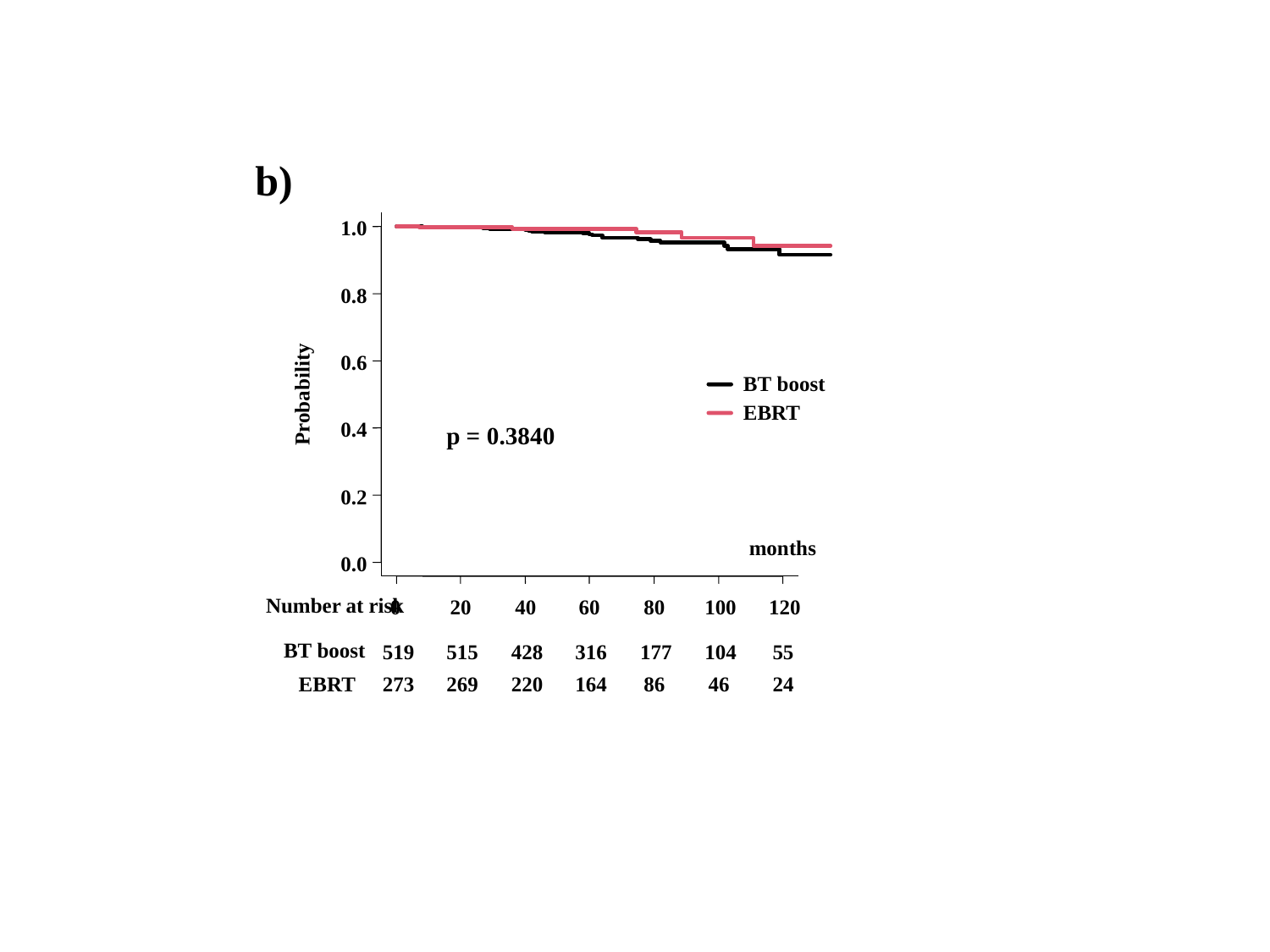

b)
1.0
0.8
0.6
BT boost
Probability
EBRT
p = 0.3840
0.4
0.2
months
0.0
Number at risk
0
20
40
60
80
100
120
BT boost
519
515
428
316
177
104
55
EBRT
273
269
220
164
86
46
24

## Slide 3
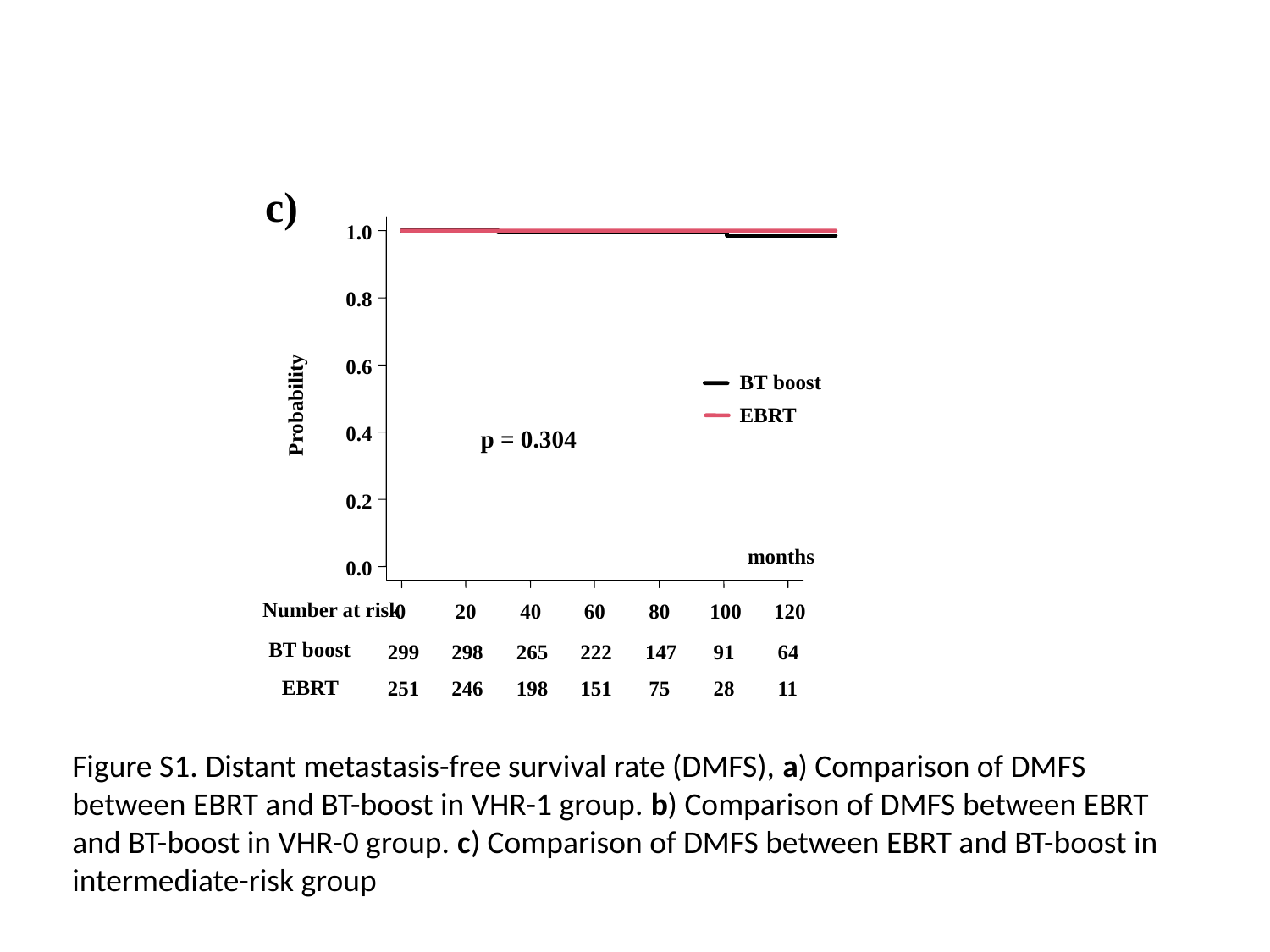

c)
1.0
0.8
0.6
BT boost
Probability
EBRT
p = 0.304
0.4
0.2
months
0.0
Number at risk
0
20
40
60
80
100
120
BT boost
299
298
265
222
147
91
64
EBRT
251
246
198
151
75
28
11
Figure S1. Distant metastasis-free survival rate (DMFS), a) Comparison of DMFS between EBRT and BT-boost in VHR-1 group. b) Comparison of DMFS between EBRT and BT-boost in VHR-0 group. c) Comparison of DMFS between EBRT and BT-boost in intermediate-risk group

## Slide 4
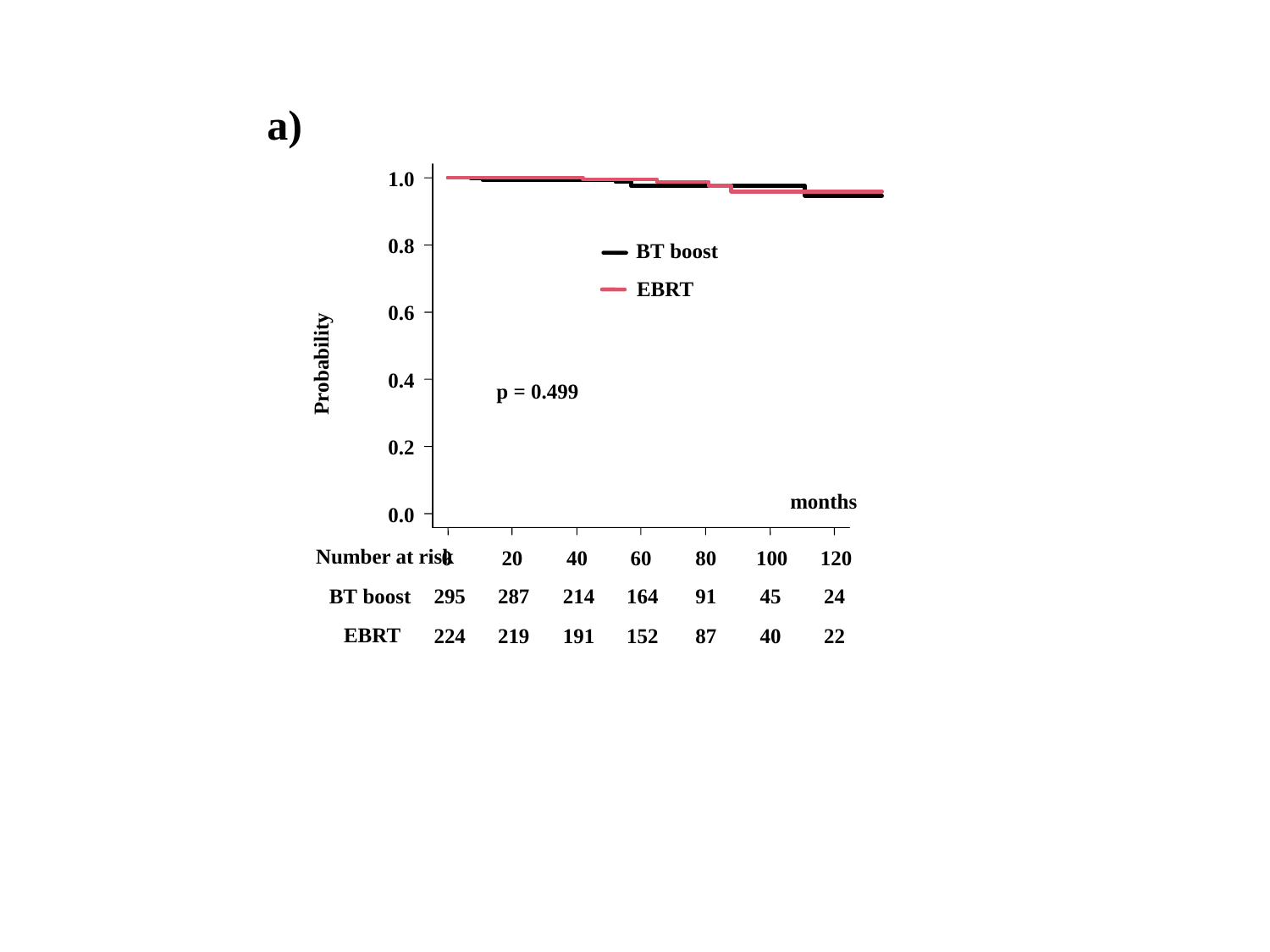

a)
1.0
0.8
BT boost
EBRT
0.6
Probability
0.4
p = 0.499
0.2
months
0.0
0
20
40
60
80
100
120
BT boost
295
287
214
164
91
45
24
EBRT
224
219
191
152
87
40
22
Number at risk

## Slide 5
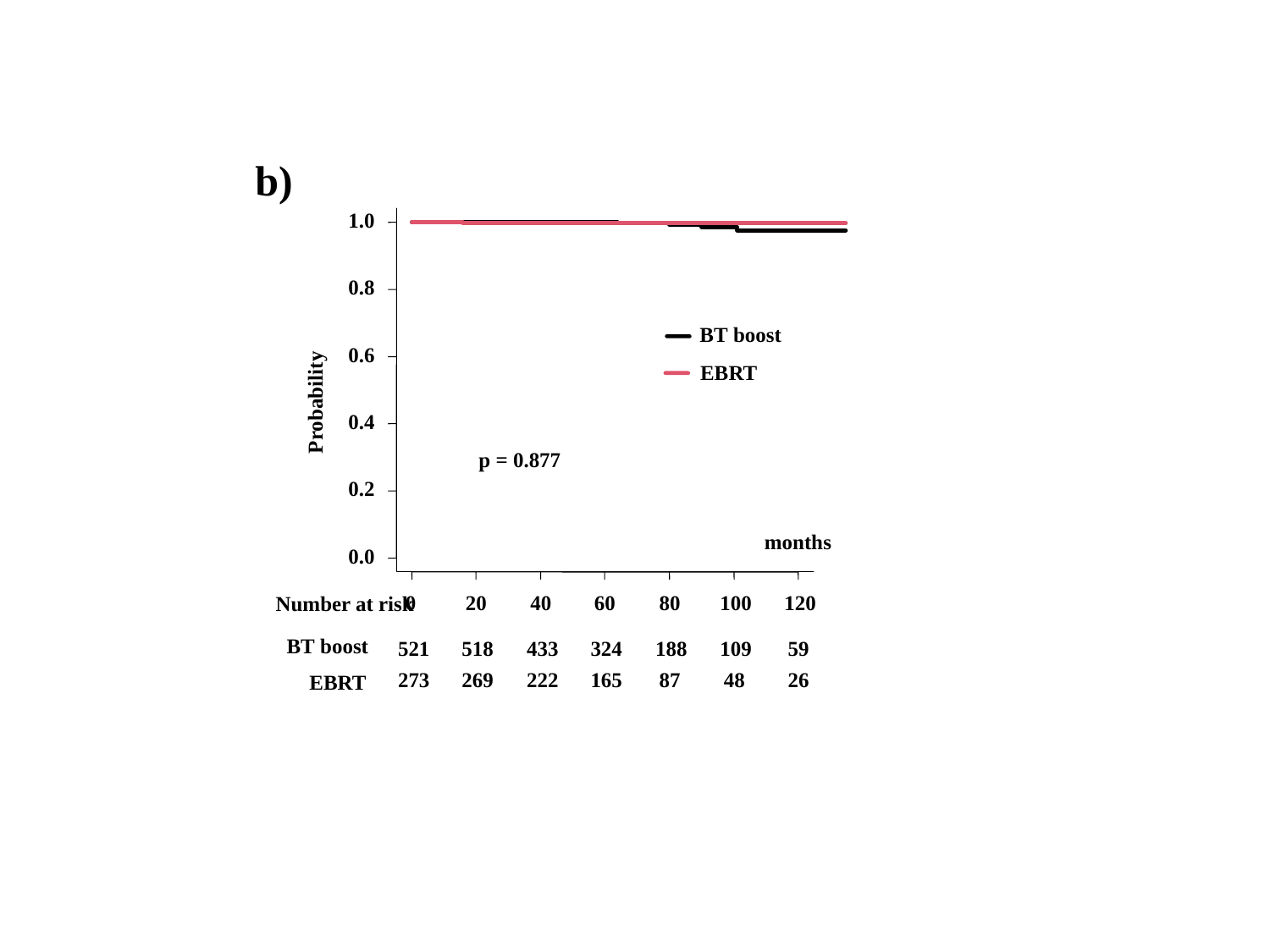

b)
1.0
0.8
BT boost
0.6
EBRT
Probability
0.4
p = 0.877
0.2
months
0.0
0
20
40
60
80
100
120
BT boost
521
518
433
324
188
109
59
273
269
222
165
87
48
26
EBRT
Number at risk

## Slide 6
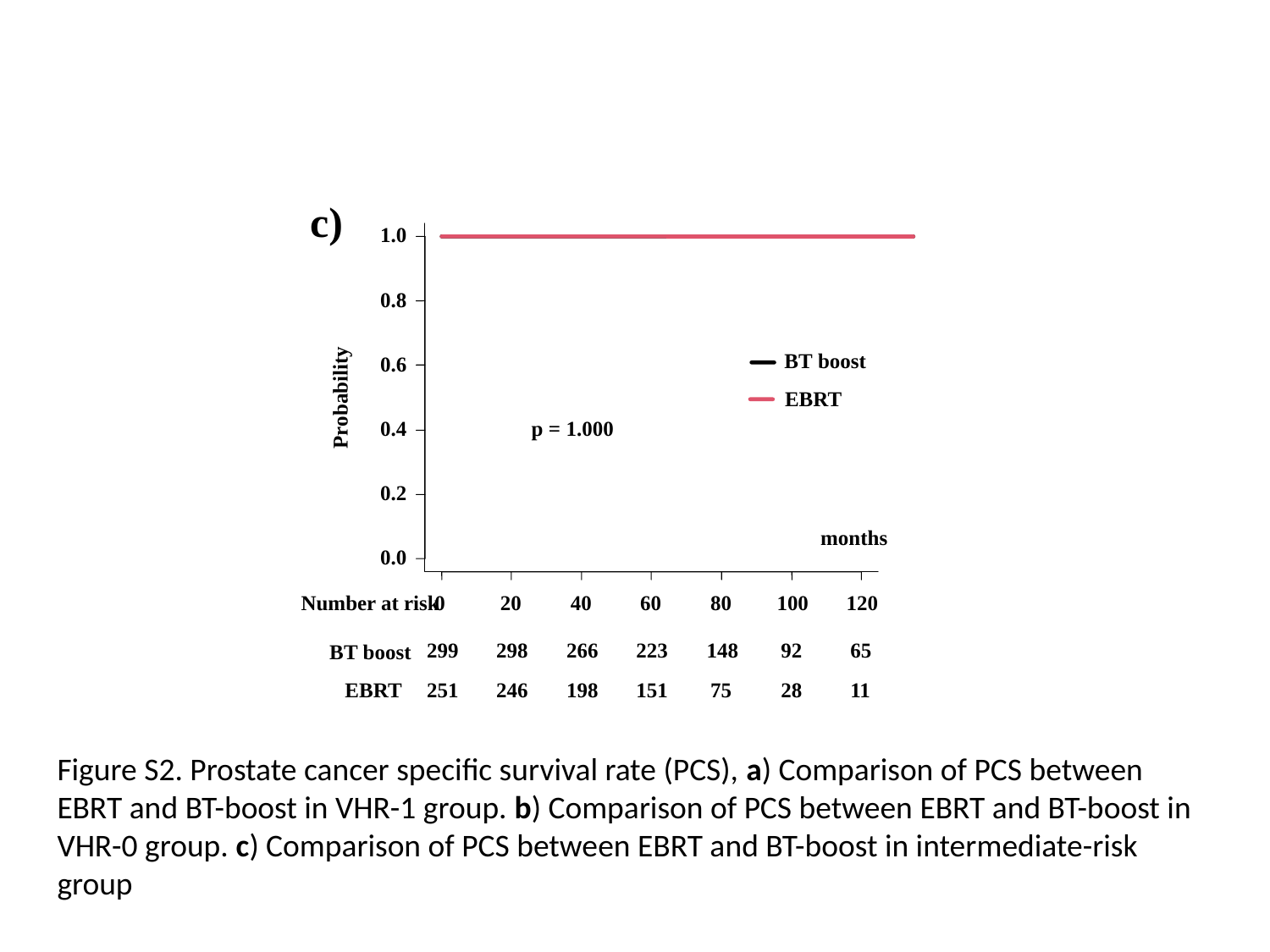

c)
1.0
0.8
BT boost
0.6
Probability
EBRT
p = 1.000
0.4
0.2
months
0.0
0
20
40
60
80
100
120
299
298
266
223
148
92
65
BT boost
EBRT
251
246
198
151
75
28
11
Number at risk
Figure S2. Prostate cancer specific survival rate (PCS), a) Comparison of PCS between EBRT and BT-boost in VHR-1 group. b) Comparison of PCS between EBRT and BT-boost in VHR-0 group. c) Comparison of PCS between EBRT and BT-boost in intermediate-risk group

## Slide 7
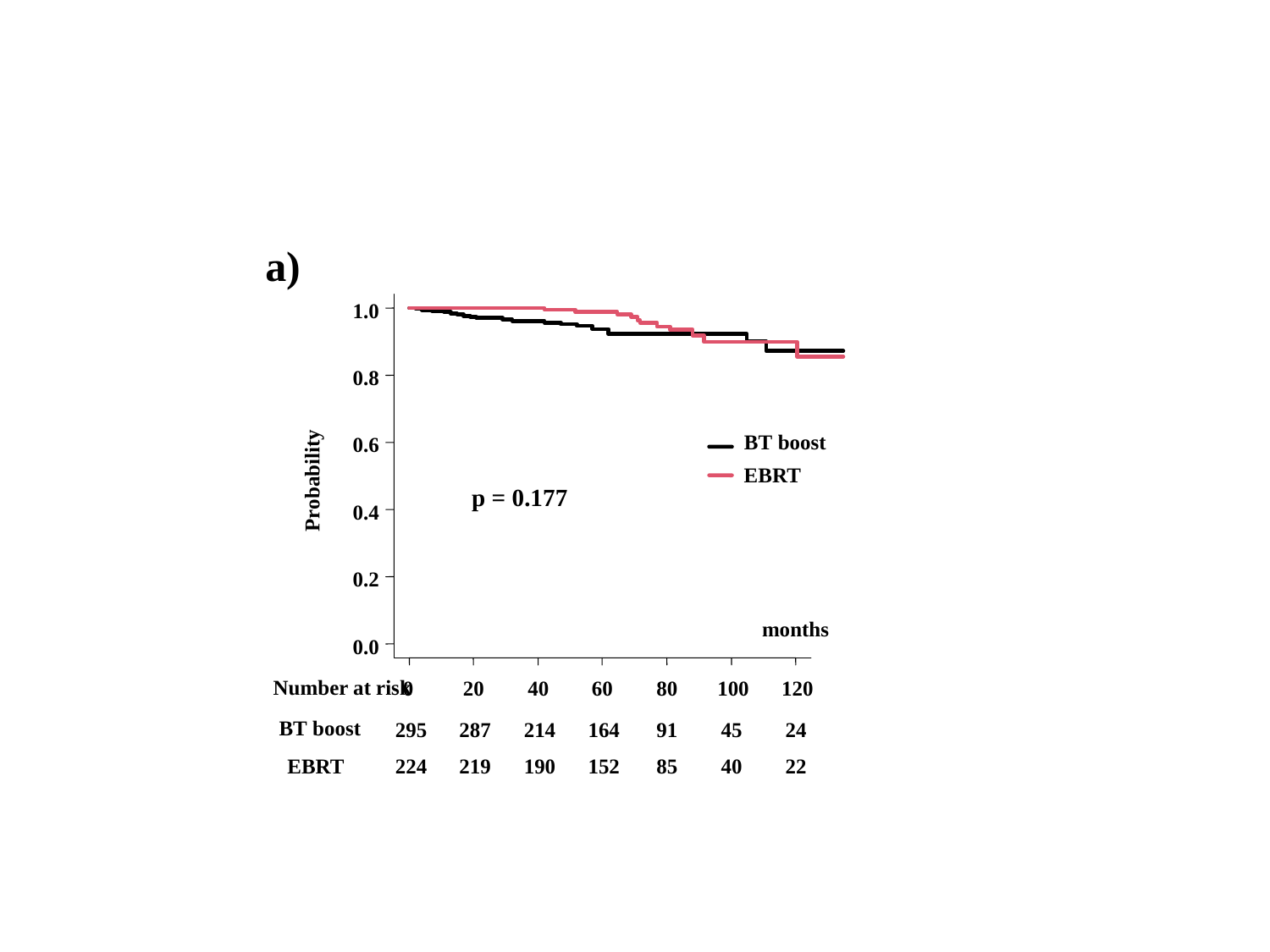

a)
1.0
0.8
BT boost
0.6
EBRT
Probability
p = 0.177
0.4
0.2
months
0.0
Number at risk
0
20
40
60
80
100
120
BT boost
295
287
214
164
91
45
24
EBRT
224
219
190
152
85
40
22

## Slide 8
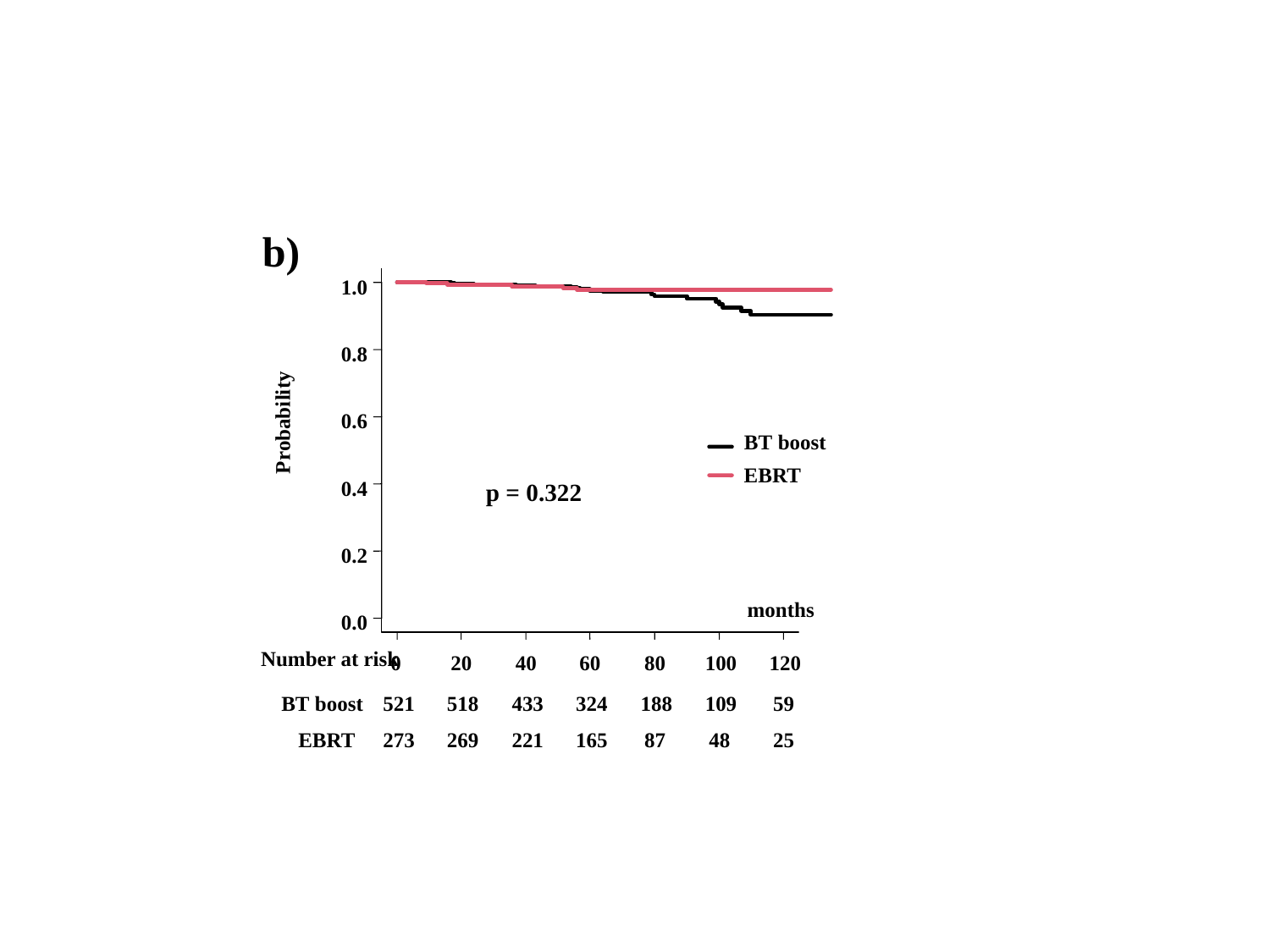

b)
1.0
0.8
0.6
Probability
BT boost
EBRT
p = 0.322
0.4
0.2
months
0.0
Number at risk
0
20
40
60
80
100
120
BT boost
521
518
433
324
188
109
59
EBRT
273
269
221
165
87
48
25

## Slide 9
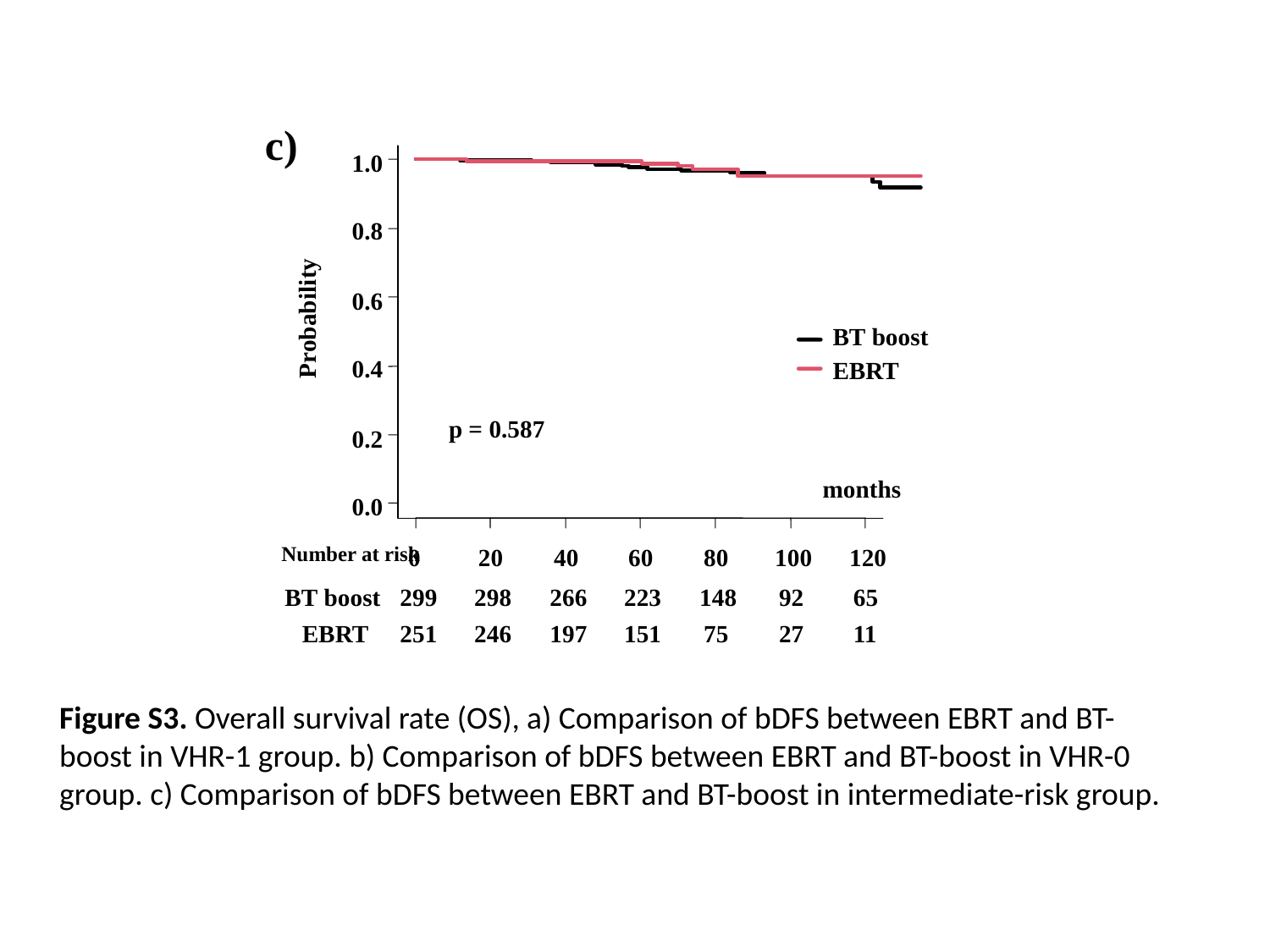

c)
1.0
0.8
0.6
Probability
BT boost
0.4
EBRT
p = 0.587
0.2
months
0.0
0
20
40
60
80
100
120
BT boost
299
298
266
223
148
92
65
EBRT
251
246
197
151
75
27
11
Number at risk
Figure S3. Overall survival rate (OS), a) Comparison of bDFS between EBRT and BT-boost in VHR-1 group. b) Comparison of bDFS between EBRT and BT-boost in VHR-0 group. c) Comparison of bDFS between EBRT and BT-boost in intermediate-risk group.
